# Amantadine for Traumatic Brain Injury—Supporting Evidence and Mode of Action

**DOI:** 10.3390/biomedicines12071558

**Published:** 2024-07-13

**Authors:** Andrzej Dekundy, Gerald Pichler, Reda El Badry, Astrid Scheschonka, Wojciech Danysz

**Affiliations:** 1 Merz Therapeutics GmbH, Eckenheimer Landstraße 100, 60318 Frankfurt am Main, Germany; andrzej.dekundy@merz.de (A.D.); astrid.scheschonka@merz.de (A.S.); 2 Department of Neurology, Albert-Schweitzer-Hospital Graz, Albert-Schweitzer-Gasse 36, 8020 Graz, Austria; gerald.pichler@stadt.graz.at; 3 Department of Neurology and Psychiatry, Faculty of Medicine, Assiut University Hospital, Assiut University, Assiut 71526, Egypt; redaalbadry02@gmail.com; 4 Danysz Pharmacology Consulting, Vor den Gärten 16, 61130 Nidderau, Germany

**Keywords:** amantadine, traumatic brain injury, clinical, preclinical, mechanism of action, sigma-1, aromatic amino acids decarboxylase, GDNF, NMDA receptors, in vivo, in vitro

## Abstract

Traumatic brain injury (TBI) is an important global clinical issue, requiring not only prevention but also effective treatment. Following TBI, diverse parallel and intertwined pathological mechanisms affecting biochemical, neurochemical, and inflammatory pathways can have a severe impact on the patient’s quality of life. The current review summarizes the evidence for the utility of amantadine in TBI in connection to its mechanism of action. Amantadine, the drug combining multiple mechanisms of action, may offer both neuroprotective and neuroactivating effects in TBI patients. Indeed, the use of amantadine in TBI has been encouraged by several clinical practice guidelines/recommendations. Amantadine is also available as an infusion, which may be of particular benefit in unconscious patients with TBI due to immediate delivery to the central nervous system and the possibility of precise dosing. In other situations, orally administered amantadine may be used. There are several questions that remain to be addressed: can amantadine be effective in disorders of consciousness requiring long-term treatment and in combination with drugs approved for the treatment of TBI? Do the observed beneficial effects of amantadine extend to disorders of consciousness due to factors other than TBI? Well-controlled clinical studies are warranted to ultimately confirm its utility in the TBI and provide answers to these questions.

## 1. Selected Epidemiological Aspects of Traumatic Brain Injury

Traumatic brain injury (TBI) is one of the leading causes of death and disabilities, ranging from paralysis to a plethora of psychiatric abnormalities. It is most often caused by vehicle accidents and falls. In the USA, based on the Centers for Disease Control and Prevention report for 2014, TBI contributed to nearly 3 million emergency department visits and hospitalizations [1]. Annually, an estimated 200,000 individuals who have sustained TBI need hospitalization. TBI leads to 56,000 deaths and was reported to account for approximately 40% of all deaths from acute injuries in the USA [1,2]. The mortality rate was found to be high (33%) in severe TBI, while it was much lower (2.5%) in moderate TBI [2].

In Europe, TBI incidence amounts to 500 cases per 100,000 population [3]. In a more recent, extensive review, including sixty-six studies from European countries, Brazinova et al. reported crude incidence rates ranging from 47.3 to 694/100,000 persons/year (country-level studies) and 83.3 to 849/100,000/year (regional-level studies) [4]. Crude mortality rates ranged from 9 to 28.10/100,000/year (country level) and 3.3 to 24.4/100,000/year (regional level). Similar to the USA, the most common reasons for injury were traffic accidents and falls [4]. Majdan et al. [5] reported the occurrence of a total of 17,049 TBI-related deaths (translating into 374,636 years of lost lives, YLLs) in 16 European countries in the year 2013. The pooled age-standardized rate of YLLs per 100,000 people per year was 259.1 [6]. The same research group estimated that in the year 2012, in the European Union (approx. 500 million), there were roughly 57,000 TBI-related deaths and 1,000,000 hospital discharges. At the same time, in the entirety of Europe (approx. 750 million), approximately 82,000 deaths and about 2,100,000 hospital discharges occurred. The authors concluded that TBI is an important cause of death and hospital admissions in Europe [5]. In summary, even though epidemiological data vary across different geographical regions, TBI remains a very relevant clinical issue deserving a great deal of attention about both prevention measures and treatment all over the world.

## 2. Pathophysiology of Traumatic Brain Injury and Possible Therapeutic Window for Intervention with Amantadine

**Figure 1:**
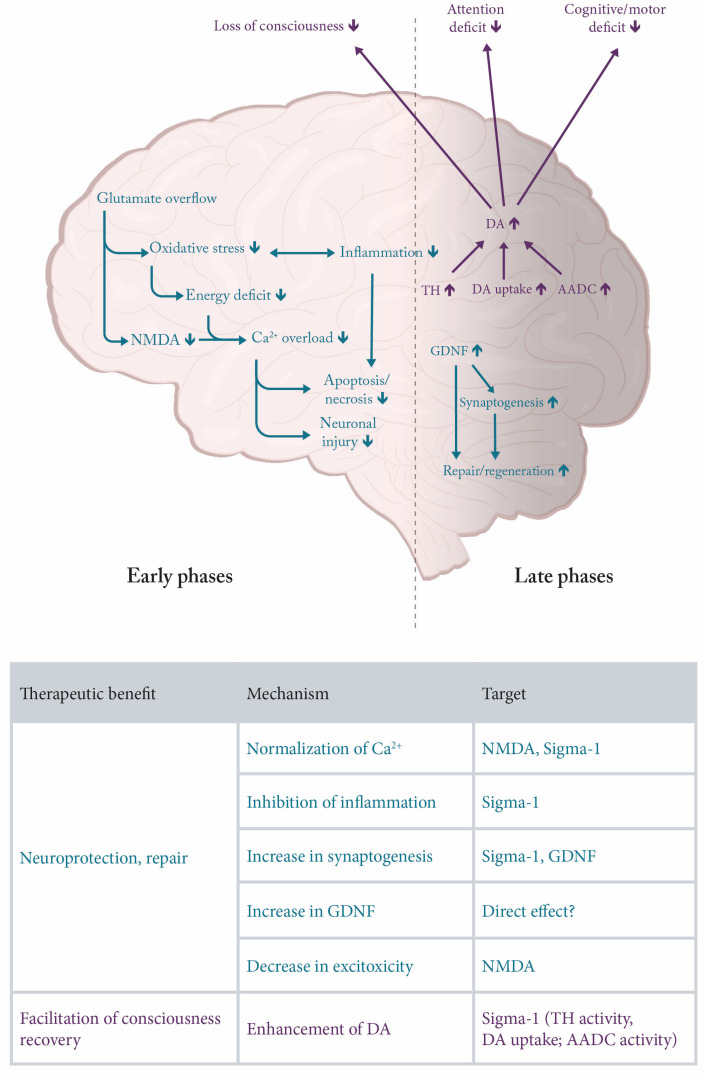
Pathophysiology of TBI and possible targets of amantadine. AADC—aromatic l-amino acids decarboxylase; Ca^2+^—calcium cation; DA—dopamine; GDNF—glial cell line-derived neurotrophic factor; NMDA—N-methyl-D-aspartate; TH—tyrosine hydroxylase.

There are diverse causes of primary damage in TBI, but the mechanisms of subsequent damage underlying the pathology and observed symptoms seem to converge (Figure 1). While there is only limited knowledge about specific causal mechanisms underlying TBI, there is accumulating evidence that interplay between oxidative stress, excitotoxicity, inflammation, lysosomal and autophagy dysfunction, etc., are key elements leading to cell death [7,8,9]. It is not likely to find a treatment halting cell death, but the therapeutic benefit resulting from the inhibition of secondary degeneration and/or improving recovery and functional outcomes is more realistic.

According to severity, TBI can be categorized as mild, moderate, or severe [10]. In mild TBI, 30–53% of patients show disability symptoms for at least one year, but their life expectancy is typically unchanged [10]. In contrast, moderate to severe TBI is connected with a progressive loss of consciousness, as well as cognitive and neurological impairments [11].

In terms of the temporal sequence, both pathomechanisms and symptoms of TBI can be categorized into those that are characteristic of the acute phase (hours), the subacute phase (days to weeks), and the chronic phase (weeks to years). Each of these three phases is characterized by the presence of distinct mechanisms and symptoms [9] (Figure 1).

Amantadine can positively influence chronologically different, sometimes cascading damage and recovery processes taking place after a TBI due to its diverse modes of action (Figure 1). During the initial acute phase of TBI, the window for therapeutic intervention is very narrow, making a pharmacological therapeutic intervention, e.g., with amantadine, virtually impossible. However, the subacute phase, in which additional secondary neurodegenerative processes occur, offers a potential therapeutic window to interrupt some of the ongoing damaging processes; in this phase, beneficial effects of amantadine on TBI-induced pathology might be expected (Figure 1). Also, in the chronic stage of TBI, which may involve cognitive deficits, affective alterations, sleep disturbances, and aggression [11], amantadine may be useful by providing support for dopaminergic transmission (Figure 1).

## 3. Damage and Subsequent Dysfunction in Traumatic Brain Injury

The primary damage results from a direct impact of a force that can lead to different, multiple sequelae, including skull fractures and intracranial bleeding (coup mechanism); it can also occur in the contrecoup contusion mechanism, whereby injury takes place at a brain area opposite to the side of the directly impacted area. The initial damage in TBI may also occur due to diffuse axonal injury in which widespread lesions are present in both white and grey matter because of the effect of forces associated with rapid acceleration and/or deceleration (e.g., in traffic accidents or falls). For a thorough review, see, e.g., McKee and Daneshvar, 2015 [12]. Due to the nature of TBI’s causes and the very short duration of the phase of primary damage, the window for a pharmacological intervention at this stage is very short-lasting and thus extremely challenging.

There are multiple mechanisms possibly involved in secondary damage in TBI. Cerebral metabolic dysfunction may result directly from the primary injury or occur in the course of any secondary processes. This dysfunction can lead to reduced cerebral metabolism (as measured by oxygen and glucose consumption) and to a reduced energetic status of the brain [13].

In terms of cerebral autoregulation, a change in cerebral perfusion pressure leads to either vasoconstriction or vasodilation. When this autoregulation mechanism gets depleted due to a TBI, there is a risk of secondary ischemia. This can also occur as a response to hypo- or hypercapnia and is referred to as cerebrovascular CO_2_ reactivity [14]. When the intracerebral pressure increases and reaches the value of the mean systolic blood pressure, the cerebral blood flow decreases. As a result, the systemic blood pressure increases, and the cerebral vessels expand. Consequently, the intracerebral pressure increases even further, which is followed by cerebral hypoxia and cerebral edema (sometimes associated with herniation) [15,16]. In vasogenic cerebral edema, reflexive dilation of the brain vessels and a mechanical–functional disturbance of the endothelial wall both lead to a disruption of the blood–brain barrier and an accumulation of a relatively large volume of fluid in the extracellular space. In the cytotoxic (intracellular) brain edema, it is the altered permeability of the cell membrane that leads to altered reabsorption of osmotically active substances and thus to a change in the cellular osmolality. The associated intracellular water accumulation primarily affects neurons, microglia, and astrocytes [17].

Minutes after a TBI, extracellular levels of the excitatory amino acids glutamate and aspartate rise dramatically [18]. This leads to excessive stimulation of the N-methyl-D-aspartate (NMDA) receptors, leading to the depolarization of neurons. The increased glutamate outflow results in an increased Na^+^ and Ca^2+^ influx into the cell and eventually leads to triggering cell damage mechanisms by Ca^2+^ overload. This occurs first in neurons, while astrocytes can take up glutamate and convert it into glutamine. The resulting increase in activity of the Na^+^/K^+^-ATPase raises the metabolic demand. The magnitude of glutamate release is age-dependent, being more pronounced in older TBI patients than in younger ones [19]. The widely described NMDA receptor antagonist properties of amantadine may potentially contribute to the observed beneficial effects of this compound in TBI patients (for details, refer to Section 5.1).

After a TBI, stimulation of the NMDA receptor leads to the release of glutamate and, ultimately, to intracellular accumulation of Ca^2+^ in the mitochondria. The most important consequence of the increased Ca^2+^ load is the formation of a mitochondrial permeability transition pore, which ultimately leads to emptying of the Ca^2+^ pool into the cytoplasm. In turn, this paves the way for apoptosis [20]. As for excitotoxicity, also for this pathway, the amantadine’s NMDA receptor antagonism may contribute to its reported therapeutic effects. It should, however, be mentioned that the pharmacological profile of amantadine extends beyond NMDA receptor blockade; a detailed discussion of putative amantadine targets can be found in Section 5.

The oxidative stress is caused by the imbalance between the production of free radicals and the body’s ability to neutralize their harmful effects through endogenous antioxidant mechanisms. The depletion of endogenous antioxidants (e.g., superoxide dismutase, glutathione peroxidase, catalase) leads to the excessive production of reactive oxygen species and related species (nitric oxide, superoxide, and hydrogen peroxide). Free radicals have unpaired electrons and find them in the environment, thus leading to the oxidation of proteins, the cleavage of DNA, and the inhibition of the mitochondrial electron transport chain. This, in turn, leads to inflammatory processes, immediate cell death, or triggers delayed apoptotic programs [21]. Indeed, there is some limited preclinical evidence existing for amantadine’s antioxidant properties (cf. Section 5.5).

TBI leads to immunological and inflammatory tissue reactions. Inflammation can cause damage on one hand and promote regeneration on the other. In the acute phase, the direct injury, being a consequence of a mechanical impact, is accompanied by disruption of the blood–brain barrier and inflammation involving the release of cytokines and mobilization of neutrophils and macrophages [9]. However, during the subacute phase, additional secondary neurodegenerative processes occur, which are accompanied by apoptosis and increasing immune response with activation of microglia (promoting phagocytosis and delivering growth factors to the injured tissue), astrocytes, as well as T and B lymphocytes [9]. Microglia also deliver growth factors to injured brain tissue. This stage offers a potential therapeutic window to interrupt some of the ongoing damaging processes. In this phase, beneficial effects of amantadine on humoral and cellular TBI-induced pathology might be expected (Figure 1).

Both primary and secondary injuries activate the release of cellular mediators (cytokines, prostaglandins, free radicals, and complement) [22]. Leukocytes, macrophages, and T-cell lymphocytes infiltrate injured tissue, which is degraded in response to these inflammatory processes. Additionally, within hours of injury, pro-inflammatory enzymes and other mediators (e.g., tumor necrosis factor [TNF] and interleukin IL-1-ß and IL-6) are upregulated. Amantadine may exert anti-inflammatory effects mediated through the inhibition of microglial activation and inflammatory cytokines such as interferons and TNF, as well as through stimulation of IL production [23,24,25]. All of the aforementioned factors can influence the inflammatory response characteristic for the pathophysiology of TBI.

Neuronal cell death following TBI causes neurological deficits and mortality. Neuronal death phenotypes are categorized based on morphological or molecular changes. In necrosis—a passive process—a loss of ionic homeostasis, failure of membrane integrity, and swelling of organelle and cells take place. On the other hand, apoptosis is an active, energy-dependent process of condensation and fragmentation of the cytoplasm and the nucleus, leading to a decrease in cell volume with a preserved structure of the organelle. In some cases, apoptosis and necrosis coexist, constituting an intermediate type of cell death, sometimes referred to as aponecrosis. It has been suggested that future neuroprotective strategies need to target multiple pathways to reflect both regional and temporal changes underlying different types of neuronal cell death (for a review, see [26] and the references therein). Indeed, some experimental studies suggest that the anti-necrotic, anti-apoptotic, and neuroprotective effects of amantadine could be related to its antioxidant, anti-inflammatory, and biochemical mechanisms [27,28,29].

## 4. Disorders of Consciousness—Recovery Enhancement

Consciousness is thought to comprise arousal (wakefulness, sustained attention, and vigilance) and awareness (subjective perceptions, feelings, thoughts) [30,31]. Arousal and vigilance require normal function of the brainstem and the thalamus [32,33,34,35], which are interrelated with the parts of the frontoparietal network known to be impaired in subjects presenting with disturbances of consciousness [36,37,38]. Dopamine is a neurotransmitter most implicated in arousal and consequently also in the TBI; indeed, widespread axonal injury is related to reduced brain dopamine availability [39,40].

Coma has been described as a pathological state characterized by severe and prolonged dysfunction of vigilance and consciousness [41] and may either occur due to a diffuse insult to both hemispheres (e.g., epileptic seizures, poisoning, or drug or alcohol overdose) or due to a focal insult (e.g., stroke or head trauma) [42].

While a subset of comatose patients presents with an extensive or complete recovery of awareness, many others who awaken from the acute comatose state do not show any signs of awareness. If repeated examinations yield no evidence of a sustained, reproducible, purposeful, or voluntary behavioral response to visual, auditory, tactile, or noxious stimuli, a diagnosis of an “unresponsive wakefulness syndrome” is made one month after the injury [43]. Some patients remain in this condition. Others eventually show inconsistent but reproducible signs of awareness, including the ability to follow commands, but they remain unable to communicate interactively. In 2002, the Aspen Neurobehavioral Conference Work Group coined the term “minimally conscious state” (MCS) to describe the condition of such patients, thereby adding a new clinical entity to the spectrum of disorders of consciousness [44]. The MCS diagnosis has been further sub-categorized into MCS minus and MCS plus. The most frequent signs of consciousness in MCS minus patients are visual fixation and pursuit, automatic motor reactions (e.g., scratching, pulling the bed sheet), and localization to noxious stimulation, whereas MCS plus patients can, in addition, follow simple commands, intelligibly verbalize, or intentionally communicate [45].

The most frequent cause of an unresponsive wakefulness syndrome in Western countries is cerebral hypoxia after cardiopulmonary resuscitation [46]. The prevalence of unresponsive wakefulness syndrome at six months after TBI has not significantly changed over the past four decades [47].

There is now convincing evidence for the use of amantadine in disorders of consciousness, in addition to conventional stimulation methods [44,48,49].

## 5. Potential Mechanism of Amantadine Effects in Traumatic Brain Injury—NMDA Receptors and Beyond

Recently, we analyzed possible targets of amantadine that could play a role in its observed therapeutic effects based on a comparison of its concentrations reached at a putative target in humans following the administration of this drug at therapeutic doses and in vitro affinity at this target [50]. The analysis demonstrated that several targets, such as sigma-1 receptors, aromatic l-amino acids decarboxylase (AADC), and glial cell line-derived neurotrophic factor (GDNF), were found to possibly have stronger involvement in amantadine’s actions than the glutamatergic NMDA receptors. For the purpose of that analysis, we also considered that intracellular concentrations of amantadine in the brain are 10 or 20 times higher than plasma levels in animal and human studies, respectively, due to lysosomal trapping [51,52,53]. Although there are dozens of publications showing in vitro inhibition of NMDA receptors by amantadine, only one of them observed that effect at a therapeutic range of concentrations (up to 10 µM) [50].

Below, we provide a short characterization of the most relevant therapeutic targets with evidence supporting their potential utility in TBI treatment.

### 5.1. NMDA Receptors and Neuroprotection

As discussed in a recent review, there are many functional and binding studies showing the inhibition of glutamatergic NMDA receptors in a range from 10 µM to 640 µM [50]; however, only one of these studies showed this effect at maximal plasma concentrations achieved at therapeutic doses (approximately 10 µM). It cannot, however, be excluded that an incomplete inhibitory effect on NMDA receptors may be supportive of other mechanisms, which are described in the following (see also Figure 1).

Shortly after the discovery of NMDA receptors [54,55] and their high permeability to calcium, their role in acute and chronic brain insult has been postulated [56,57]. However, all clinical trials with NMDA receptor antagonists in stroke or TBI failed [58], likely due to a need to administer these substances at doses that may actually produce a detrimental effect on neuronal recovery. Therefore, NMDA antagonism alone cannot be regarded as a viable approach to prevent TBI-induced damage, but it could still support other mechanisms if the degree of NMDA receptor blockade remains mild to moderate. We believe that this may be the case for amantadine, which produces only a weak effect on NMDA receptors at therapeutic doses (see also [50]).

It has recently been suggested that improved neuroprotective effects can be achieved by selective targeting of extrasynaptic NMDA receptors of the so-called “death signaling complex”. These receptors are mainly composed of NR2B subunits and coupled to different signaling pathways than the physiologically more relevant subsynaptic receptors [59,60]. It is, however, not known whether amantadine has a preference for these extrasynaptic receptors.

It is beyond the scope of this review to discuss all studies focusing on the effects of NMDA receptor antagonists in animal models of TBI. It should, however, be mentioned that the majority of these studies showed beneficial effects in terms of improvement in structural and/or functional outcomes, as reviewed elsewhere in more detail [61,62,63,64].

As mentioned above, the positive preclinical data did not result in a therapeutic use of such compounds since clinical studies failed to demonstrate their efficacy [58].

### 5.2. Sigma 1 Receptors and Neuroprotection

Kornhuber and colleagues were the first to describe that amantadine binds to sigma-1 receptors with approximately 20 µM of Ki, as evidenced by [^3^H](+)-pentazocine binding in homogenates of post mortem human frontal cortex [65]. Even a higher affinity was observed in guinea pig or rat brain homogenates [66,67]. Amantadine seems to function as an agonist at sigma-1 receptors [67].

These receptors are located intracellularly on membranes of the endoplasmatic reticulum and mitochondria, where they control Ca^2+^ signaling [68,69,70].

There are many studies indicating the involvement of sigma-1 receptors in the function of the dopaminergic system, which may have implications for the effect of amantadine on recovery from TBI, in particular for a faster return to the conscious state (Figure 1). Sigma-1 receptor activation enhances tyrosine hydroxylase (TH) activity [71], increases dopamine in vivo in the striatum [72], and decreases DA uptake [73]. Moreover, it has been described that sigma-1 ligands modulate NMDA-stimulated dopamine release [74].

Apart from their role in the modulation of dopamine transmission, sigma-1 receptors have been associated with neuroprotective activity, which has been demonstrated in various models focusing on neuronal insults [75,76,77,78,79,80,81,82]. Studies in animal models of neurodegenerative diseases, reviewed recently by Shi and colleagues [83], indirectly support amantadine’s use in TBI.

How can the neuroprotective effect of sigma-1 agonism be mediated? It has been suggested that upon ligand stimulation, the sigma-1 receptor dissociates from the binding immunoglobulin protein on the endoplasmic reticulum (ER) membrane and modulates three sensors of ER stress. These comprise protein kinase RNA-like ER kinase, inositol requiring enzyme 1α, and factor 6 [83]. Similar protective mechanisms occur in mitochondria, which play a crucial role in TBI. A change of balance between anti-apoptotic/pro-apoptotic factors and reactive oxygen species is part of these mechanisms.

Sigma-1 receptors have been suggested to exert a dual effect on NMDA receptors. They enhance the function of synaptic NMDA receptors responsible for plasticity while they inhibit extrasynaptic NMDA receptors responsible for excitotoxic neuronal death [83].

Several effects, such as a decrease in ER stress, improvement in mitochondrial function, normalization of calcium homeostasis, and inhibition of excitotoxicity, could play in concert with recovery from TBI [83].

On top of that, improvement in recovery may be supported by the inhibition of microglia-mediated inflammation, including the normalization of an imbalance of M1/M2 phenotypes. These subpopulations have pro- and anti-inflammatory functions, respectively [83,84].

The data on the efficacy of sigma-1 ligands in animal models of TBI are limited. In one study, the activation of sigma-1 by 2-(4-morpholinethyl)-1-phenylcyclohexanecarboxylate (PRE-084, 10 mg/kg i.p.) given 15 min after TBI reduced the lesion volume, brain edema, neurological severity score, and accelerated body weight recovery [85]. A decrease in microglia activation was also observed.

The activation of sigma-1 receptors is important to ensure the anti-inflammatory effect of amantadine [85,86].

In summary, it may be expected that sigma-1 receptor activation may enhance recovery from TBI (Figure 1) through increased synaptogenesis and inhibition of inflammation [83,85].

To the best of our knowledge, there have been no clinical trials with selective sigma-1 ligands in TBI.

### 5.3. Aromatic Amino Acids Decarboxylase and Neuroactivation

Amantadine was demonstrated to increase the activity of AADC, the enzyme responsible for dopamine synthesis [87]. In this way, dopaminergic activity increases, which can have a supportive effect on recovery after TBI since dysfunctions of the dopaminergic and noradrenergic systems occur here.

In vitro, in pheochromocytoma (PC12) cells, amantadine (at 10 µM) enhances the expression of mRNA of AADC by 70% [88]. In an ex vivo study in rats, amantadine (at 40 mg/kg) increased the activity of AADC in the striatum (3-fold) and in the substantia nigra (10-fold) one hour after injection [89].

Amantadine (30 mg/kg) administered to rats subjected to 6-hydroxydopamine (6-OHDA) lesions of the dopaminergic system increases ex vivo L-DOPA conversion in the striatum, indicating increased AADC activity [90].

In humans, Deep and colleagues [87] showed that amantadine (100 mg for 3 days) increases the activity of AADC up to 27% in the ventral striatum using 6-[^18^F]fluoro-L-DOPA (L-DOPA = 3,4-Dihydroxy-L-phenylalanin) as an exogenous AADC substrate.

Enhanced activity and/or an increase in concentration leads to an increase in dopamine levels, which can be released to the synaptic cleft. In turn, this effect could be clearly supportive of recovery from TBI (Figure 1), in particular for enhancement of recovery from unconsciousness and cognitive performance [91,92,93].

In TBI, the benefit of dopamine enhancement is not expected in the initial insult [39,94]. This relates to the fact that excess dopamine produces oxidative stress and energy deficit and activates inflammation [39,94]. At the same time, dopaminergic neurons are victims of neurotoxicity in the hippocampus and striatum, resulting in the impairment of cognitive and motor function, respectively [39,94]. In the chronic phase, this creates a gradually increasing dopaminergic deficit in the aforementioned structures. In turn, the enhancement of dopaminergic transmission may be particularly useful to enhance and/or increase the recovery of cognitive and motor functions. Apart from amantadine, positive effects in TBI have been reported for enhancers of dopaminergic transmission, such as amphetamine, methylphenidate, or bromocriptine in preclinical and/or clinical conditions [39,94].

### 5.4. Glial Cell Line-Derived Neurotrophic Factor and Neuroprotection/Regeneration

The GDNF is a neurotrophin connected with action on dopaminergic neurons. It has been shown to support the neuronal morphology of these neurons and to protect against neurotoxicity through an increase in pro-survival gene expression and a decrease in pro-apoptosis factors [95]. In C6 glioma cells, amantadine, at a concentration of 5 µM, increases GDNF mRNA [96]. In primary cultures from rat midbrain, amantadine (10–30 µM) increases GDNF mRNA by up to 70% 48 and 72 h after exposure to mixed cultures of astroglia and microglia [27]. The authors indicate the role of the induction of acetylation of histone H3 by inhibiting the histone deacetylase as an underlying mechanism [27].

In rats, amantadine (25 mg/kg) increases GDNF in the hippocampus 6 and 24 h after surgery by approx. two-fold, as demonstrated by immunohistochemistry and Western blot [97]. Amantadine also improved recovery after post-operative insult. Interestingly, the attenuation of learning impairment by amantadine after surgery was inhibited by the anti-GDNF antibody [97,98], suggesting this mechanism of action.

In primary hippocampal cultures, GDNF (1 ng/mL) prevented hypoxia-induced functional and structural changes [99]. In rats with TBI, GDNF infused into the lateral ventricle for 7 days (200 ng/day) decreased neuronal loss in CA2 and CA3 hippocampal regions by approx. 50% [100]. Umbilical cord-derived mesenchymal stem cells expressing GDNF and brain-derived neurotrophic factor (BDNF) provided neuroprotection in rats subjected to TBI [101]. Similarly, AdV-GDNF delivery in a TBI model in rats enhanced neuronal survival and induced neuroprotection [102]. Supportive evidence for neuroprotective and/or restorative effects of GDNF results from studies on various models of acute and chronic neurodegenerative diseases, as reviewed recently [103,104]. Anti-inflammatory and tissue-protective functions of reactive astrocytes have been suggested to be likely mediated through GDNF [105]. In turn, the role of GDNF among other trophic factors (e.g., nerve growth factor [NGF], BDNF, basic fibroblast growth factor [bFGF], Neurotrophins-3, -4, and -5) has been implied in TBI [106]. In conclusion, the action of amantadine on GDNF may be a valuable contribution to its therapeutic effect in TBI (Figure 1).

### 5.5. Other Possible Mechanisms of Action

After chronic (6 weeks) treatment with amantadine in mice, the effectiveness of presynaptically acting CNS stimulants was reduced, while the effect of the dopaminergic agonist apomorphine was enhanced. This was accompanied by an increase in the number of spiroperidol binding to presumably dopamine receptors [107]. Also, the anti-inflammatory properties of amantadine may play a role in supporting recovery from TBI. In vitro, amantadine (4 µM) inhibited the inflammatory activation of microglia by approximately 25% following lipopolysaccharide (LPS) stimulation [23]. Moreover, at a concentration of 40 µM, amantadine protected neurons in co-culture against LPS-induced toxicity [23]. The same authors reported that in mice, amantadine (10 mg/kg) given for 4 days inhibits microglia activation and protects against 1-Methyl-4-phenyl-1,2,3,6-tetrahydropyridin (MPTP)-induced toxicity at 25 mg/kg [23]. In an in vitro study in human blood, amantadine (1 µM) inhibited the production of pro-inflammatory cytokines such as interferon-γ and tumor necrosis factor-α. [24]. Similarly, Wandinger and colleagues [25] reported that in Parkinson’s patients, amantadine correction decreased interleukine-2 and interferon-γ secretion, as measured in blood samples collected from Parkinson’s disease patients. Finally, the blockade of α4β2- and α7-nicotine receptors mediated by amantadine also appears to exert anti-inflammatory effects [108]. Furthermore, amantadine was demonstrated to exert antioxidant-like activity in vitro in the 2,2-Diphenyl-1-picrylhydrazyl (DPPH) test [28].

It has been shown that the expression of phosphodiesterase (PDE) in the hippocampus is altered after TBI. Amantadine’s suggested effect on PDE could thus favorably alter the deficits in synaptic plasticity of the hippocampus and contribute to the improvement in cognitive abilities after TBI. Amantadine inhibits calmodulin-dependent PDE 1 with an IC_50_ of approximately 5 µM, which may increase adenosine 3′,5′-cyclic monophosphate (cAMP) and, in turn, produce neuroprotective activity [109] and anti-inflammatory properties of amantadine [110]. In another in vitro study, amantadine, at a concentration of 6 µM, inhibited PDEs responsible for guanosine 3′,5′-cyclic monophosphate (cGMP) and cAMP degradation by up to 30 and 20%, respectively. This effect was even stronger, i.e., reaching 50%, when analyzed ex vivo in hemiparkinsonian rats rendered dyskinetic with repeated doses of L-DOPA. Moreover, amantadine treatment (40 mg/kg) decreased cGMP in the striatum of the dyskinetic rat brain microdialysates [111]. There is an indication that PDEs may be upregulated in TBI. The effect of amantadine on PDE could thus favorably alter the deficits in synaptic plasticity of the hippocampus and contribute to the improvement in cognitive abilities after TBI. Indeed, PDEs, particularly of group 4 (PDE4), have been suggested as potential targets for the treatment of TBI [112,113]. The reduction in PDE-1 is also related to the anti-inflammatory properties of amantadine. This shows an impact on microglia signaling pathways and the ability of PDE-1 inhibitors to prevent or attenuate an excessive inflammatory response from BV2 cells and microglia [110].

## 6. Preclinical and Clinical Evidence of Amantadine’s Efficacy in Traumatic Brain Injury

Amantadine was first developed in the 1960s as a treatment against influenza A2 [114,115]. Later, its antiparkinsonian activity was accidentally discovered by Robert Schwab [116]. Therefore, amantadine has been used for several decades in the treatment of influenza infections and Parkinson’s disease.

Amantadine’s efficacy in TBI was initially suggested by Gualtieri and colleagues based on their clinical observations [117,118]. This is probably not surprising given the fact that neuroprotection by amantadine had previously been suggested in various neurological conditions such as Parkinson’s disease, stroke, and infectious disease [119,120,121,122,123,124].

### 6.1. Preclinical Studies

A number of studies have demonstrated the neuroprotective activity of amantadine in various experimental paradigms. The effects observed in these studies can also be pertinent to the pathomechanisms of TBI. However, for the sake of keeping the focus on TBI, we refer the reader to our previous extensive review discussing these aspects [50].

Several preclinical studies were performed or have been ongoing that are specifically evaluating the effects of amantadine on different outcome measures in various animal models of TBI. From the therapeutic point of view, studies employing the delayed treatment initiation paradigm are closer to clinical practice as compared to pretreatment. The effect of amantadine may thus be related to improvement in recovery from the insult, enhancement of regeneration processes, or effect on neurochemical pathways implicated in TBI symptoms.

One of the earliest studies of amantadine in a TBI model was conducted by Dixon and colleagues in rats. The authors demonstrated that amantadine, given for 18 days, starting one day post-injury at a dose of 10 mg/kg/day, attenuated deficits in water maze learning 14–18 days after injury. The motor tasks and hippocampal histology were not improved [125].

Wang and colleagues investigated the effects of amantadine treatment initiated one hour after TBI and subsequently followed by its administration using a thrice-daily dose regimen for 16 consecutive days at 15, 45, or 135 mg/kg/day. Only the highest dose improved performance in the Morris maze spatial learning paradigm and afforded neuroprotection as observed on the level of the hippocampus. However, the effective dose resulted in serum concentrations of approximately 12,000 ng/mL (corresponding to 80 µM) [126]. Such a serum level is far above the therapeutic range of amantadine in humans.

In another study, amantadine, given at 45 or 135 mg/kg three times a day for 28 days following experimental TBI in rats, decreased the neuronal degeneration and apoptosis in the substantia nigra [127]. In addition, amantadine reversed the TBI-related decrease in dopamine in the striatum, decreased depressive-like behavior (as demonstrated in experiments using forced swim test and sucrose preference paradigms), and improved learning deficits [126,127]. Noteworthy, even the lower dose of amantadine (45 mg/kg) is expected to exceed clinically relevant plasma concentrations [50].

Bleimeister and colleagues started the administration of amantadine at a dose of 20 mg/kg to rats 24 h after cortical impact injury; the treatment was continued for 19 days. Improvement in motor and learning disabilities was observed. However, amantadine failed to improve structural changes, i.e., the volume of cortical lesions, as measured by lesion area in histological slices [128].

Huang and colleagues showed that infusion of amantadine (86.4 mg/kg/day starting five days after insult and continuing for eight weeks) reversed dopamine deficit, decreased motor impairment on the rotarod, and improved novel object recognition learning test in rats after cerebral cortical fluid percussion injury, the widely used model of brain injury [129].

In yet another study, treatment with amantadine, starting 24 h after cortical impact injury and continuing for 19 days, partially attenuated motor coordination deficit (as measured using the beam walking test on days 1–5) and improved spatial learning (on days 14–19). Interestingly, a statistically significant effect was observed at mid-dose (20 mg/kg/day) but not at 10 or 40 mg/kg/day, suggesting a bell-shaped dose–response relationship [130].

The majority of the aforementioned studies indicate some types of functional improvement by amantadine; however, most authors also report a lack of structural improvement with amantadine. The main shortcoming of many of these studies is the use of too high doses that lack therapeutic relevance. However, in general, the preclinical data clearly suggest the beneficial effect of amantadine in the post-treatment of TBI. This remains in agreement with the available favorable results of clinical studies (see below).

### 6.2. Clinical Studies

The awakening mechanism associated with amantadine in the disturbance of consciousness is related to the enhancement of dopamine in the substantia nigra and in neurotransmission within the mesencephalic limbic and frontal striatum loop system, which are responsible for regulating awakening, activation, and attention [131]. This has been confirmed by positron-emitted tomography examination [132]. Neuropharmacological therapies are commonly used off-label to enhance arousal and behavioral responsiveness on the premise that pathological derangements in dopaminergic and noradrenergic neurotransmitter systems can be improved through supplementation. In that context, amantadine is one of the most commonly used drugs. There have been a multitude of studies analyzing the effects of amantadine in recent years.

Amantadine has been widely investigated in consciousness disorders. However, the clinical trials are rather heterogeneous regarding the studied populations, treatment modalities (e.g., the timing of the initiation of the pharmacological intervention, treatment duration, the dosage), and clinical outcome measures; for review, see [133]. Indeed, both neuroprotection and neuroactivation can be envisaged as potential mechanisms underlying amantadine’s effects on overall recovery following brain injury. There is a relatively large body of evidence suggesting that amantadine promotes functional amelioration in patients following acute TBI. In the earliest published placebo-controlled randomized controlled trial (RCT) using a crossover design, amantadine failed to increase the rate of cognitive recovery in 10 patients with moderate to severe TBI [134]. A placebo-controlled RCT conducted later showed improvements with amantadine on the Disability Rating Scale (DRS) and cognitive function tests. Furthermore, following the switch to amantadine, the placebo-treated patients showed further improvements [40]. Likewise, the most robust and large placebo-controlled RCT (*N* = 184) involving patients 4–16 weeks after severe TBI in the vegetative state or minimally conscious state showed 4-week treatment with amantadine accelerated recovery as measured on the DRS and Coma Recovery Scale-Revised (CRS-R) [131]. The rate of improvement decreased during a 2-week wash-out period in the amantadine more than in the placebo group, with no difference in DRS and CRS-R scores at 6 weeks. Rates of adverse effects were similar in both groups [131].

A number of retrospective chart reviews, case–control studies, or case reports in patients with disorders of consciousness remain in concordance with the results of the aforementioned RCTs [118,135,136,137,138,139,140,141,142,143,144,145,146]. Furthermore, amantadine induced specific metabolic changes in affected brain areas of TBI patients, which were correlated with some clinical improvements [132,138]. In an open-label study, the effect of amantadine (400 mg) on executive function and activity in the pre-frontal cortex was studied in twenty-two subjects pre- and post-12-week treatment. Improvement in executive function was observed, and positron emission tomography (PET) data showed an increase in left pre-frontal cortex glucose metabolism, with a significant correlation between these two measures (Kraus et al., 2005). Shafiee et al. observed a numerical improvement in an acute phase after injury as measured with the Glasgow Coma Scale (GCS) and Glasgow Outcome Scale (GOS) with amantadine when compared with zolpidem and placebo groups, but without any significant statistical difference [147]. Very recently, Shimia et al. showed significant improvements compared to placebo on DRS, but not on GOS. The authors themselves acknowledged the limitations of their study: a small sample size, short duration, absence of a wash-out period, and shortcomings of the GOS for this kind of clinical study [148].

The therapeutic potential of amantadine has also been tested in pediatric TBI patients. In placebo-controlled studies in the pediatric population (age range of 3–18 years), amantadine was reported to be well tolerated, with an adverse effects profile similar to that of placebo [for review, see [149]. Green et al. (2004) evaluated the safety of amantadine in children with TBI, with only 5 of 54 patients experienced side effects, all of which were reversible [150]. Also, a later study investigating the effects of amantadine in pediatric TBI patients found it safe. Despite the lack of statistically significant differences in cognition, a cognition-improving potential of amantadine was suggested [151]. In yet another pediatric study—a RCT comparing amantadine to pramipexole in low-responsive children and adolescents one month after brain injury—the patients in the amantadine group made significant improvements from the baseline on several outcome parameters (Coma/Near Coma Scale, Western NeuroSensory Stimulation Profile, DRS weekly gains, and Rancho Los Amigos Scale) without any significant side effects [152]. More recently, McMahon et al. performed a randomized placebo-controlled crossover trial in children (*N* = 7). The observed improvements in consciousness parameters were greater with amantadine than with placebo. However, the differences were not found to be significant [153].

Some studies investigated the effects of amantadine on neurobehavioral parameters, e.g., irritability, aggression, or anger, in patients recovering from the TBI in its chronic phase (≥6 months following TBI). Among patients with moderate–severe irritability, amantadine significantly improved the frequency and severity of irritability and aggression and was safe [154]. Amantadine significantly reduced aggression but not anger in patients with moderate-to-severe aggression [155]. Even though aggression is one of the possible sequelae of TBI in children [149], it should be interpreted with caution, since amantadine was reported to increase aggression in pediatric TBI patients [150]. In a recent publication, McLaughlin et al. reported on amantadine use in 234 children and young adults (age range of 2 months to 21 years) with TBI during inpatient rehabilitation. Of those, 21% of patients (0.9–20 years) received amantadine. Almost half of the patients admitted with a disorder of consciousness (median age 11.6 years) were treated with amantadine (dose range of 0.7–13.5 mg/kg/d; the highest total daily dose was 400 mg/d). Nausea/abdominal discomfort (*n* = 3) and agitation (*n* = 3) were the most commonly reported adverse effects (8 patients; 16%). None of the adverse events were reported as serious [156].

To date, there are no comprehensive guidelines for the treatment of disorders of consciousness in children and adolescents. Recently, Molteni et al. [157] reviewed the available evidence with the aim of providing a base for the development of pediatric guidelines for the diagnosis, prognosis, and treatment of such disorders. Based on their analysis, amantadine treatment was associated with an improvement in consciousness parameters in approximately 55% of cases [157].

It should be mentioned that some studies failed to demonstrate favorable effects of amantadine on various outcome measures in patients with brain injury (e.g., [134,155,158,159,160,161,162]. Recently, Passman et al. evaluated the efficacy of early amantadine administration on the recovery of consciousness after severe TBI in a retrospective analysis of medical records of patients over 11 years [163]. The authors compared the patients receiving amantadine (*n* = 60) to all other patients (*N* = 344) with respect to the outcomes on GCSe, GOS-Extended score, length of stay, mortality, recovery of command following, and days to command following. The authors found no difference between these two groups in terms of mortality, rates of command following, or the percentage of patients with severe (3–8) GCS scores at discharge, but also with respect to adverse events. In addition, the amantadine group was less likely to have a favorable recovery, had a longer length of hospital stay, and a longer time to command following. The authors underlined the necessity of larger inpatient randomized trials investigating amantadine treatment for severe TBI [163].

In conclusion, there is some published evidence that amantadine improves arousal, attention, concentration, alertness, and mobility without compromising safety in comatose patients at different stages following acute brain injury [164,165]. Accordingly, amantadine has been recommended by several clinical practice guidelines related to TBI treatment [117,166,167]. It should also be mentioned that amantadine was classified by the American Academy of Neurology (AAN) at the level of evidence B in the recent guidelines for disorders of consciousness [117,166,167,168]. In addition, amantadine may have the potential to normalize behavioral disturbances in patients recovering from TBI [155]. Very recently, an expert panel (INCOG) reviewed evidence published in 2014 and developed updated guidelines for the management of attention in adults. The panel concluded that amantadine may facilitate arousal in comatose or vegetative patients but does not enhance performance on attentional measures over the longer term [169]. New evidence-based German clinical practice guidelines for the neurological rehabilitation of patients with disorders of consciousness have recently become available (Bender et al., 2023). The authors listed TBI among the most common causes of disorders of consciousness and called for the use of standardized instruments in research. Mostly based on the results of the placebo-controlled study of [131], they recommended the use of escalating doses of amantadine up to 400 mg daily to treat post-coma vigilance impairment [49].

A detailed overview of selected important clinical studies with amantadine in the indication of TBI can be found in Table 1. The table covers amantadine doses, treatment durations, study designs, descriptions of the treated population, clinical tools (e.g., scales) used, and study results.

## 7. Non-Traumatic Brain Injury

For the use of amantadine in chronic disorders of consciousness, there is also a recommendation for non-traumatic causes [49]. The authors consider this to be appropriate since the evidence for efficacy is very good, and the risk–benefit ratio speaks in favor of an application trial.

Gao et al. [173] investigated the efficacy of amantadine in non-traumatic cerebral hemorrhage. In their study, 6 out of 12 patients on amantadine regained consciousness within three months. Efficacy was lower for bleeding in the frontal, parietal, and temporal lobes than in the thalamus and basal ganglia.

No significant improvement in the recovery rate was noted in the amantadine group, but a reduction in the time to regain consciousness was reported in non-traumatic patients [174].

There is also evidence that amantadine improves attention, concentration, alertness, arousal, and mobility in comatose patients at various stages of acute brain injury [164].

## 8. Differences between Amantadine Sulfate and Hydrochloride

It should be noted that there are two amantadine salts on the market: amantadine hydrochloride, originally introduced by Dupont as Symmetrel^®^, and amantadine sulfate, introduced by Merz Pharmaceuticals as PK Merz^®^. It has been suggested that after oral treatment, the increase in plasma levels after amantadine sulfate (PK Merz^®^) is more gradual and lasts longer due to slower absorption, which is likely the result of lower solubility [175]. Due to this feature, higher doses of amantadine sulfate (up to 600 mg) have been claimed to be used with a lower risk of side effects as opposed to amantadine hydrochloride [175]. Moreover, a longer half-life provides a potential advantage of more constant plasma levels by lower treatment frequency.

**Figure 2:**
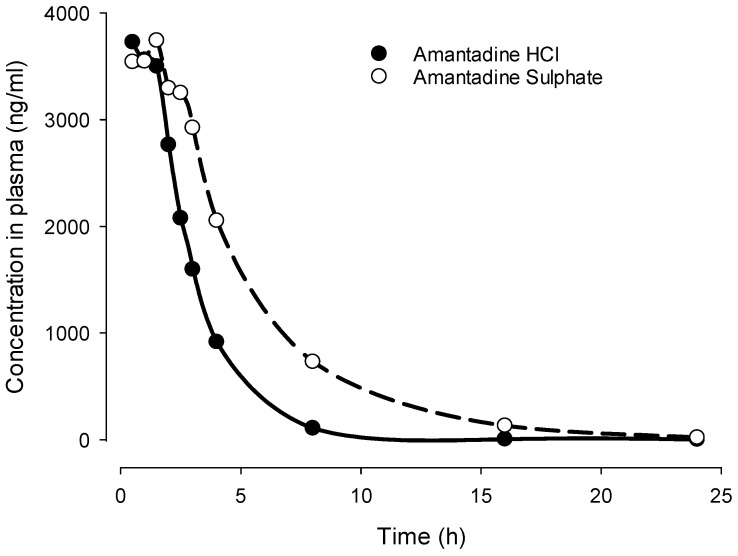
Comparison of pharmacokinetics of amantadine hydrochloride and amantadine sulfate given orally as suspension in carboxy methyl cellulose (CMC) at equimolar doses (50.00 and 53.36 mg/kg). Sulfate salt shows delayed Tmax and higher area under the curve (see Table 1 for details). Symbols are means of 8 replicates per group [176].

However, well-controlled clinical studies supporting these observations of differences between amantadine sulfate and hydrochloride are missing. Inspired by this gap, we compared the pharmacokinetics of amantadine sulfate vs. that of amantadine hydrochloride after oral administration [176] of equimolar doses to Sprague Dawley (SD) male rats using 0.5% methylcellulose as a vehicle (N = 8 per group) (Table 2, Figure 2, internal report) [176]. Plasma obtained by serial sampling was analyzed for amantadine at 0.5, 1, 1.5, 2, 2.5, 3, 4, 8, 16, and 24 h after administration using liquid chromatography–mass spectrometry (LC/MS).). Indeed, we could demonstrate that amantadine sulfate had a delayed plasma half-life (T1/2) and higher area under the curve (Table 2, Figure 2). There was also a trend for delayed Tmax, which, however, failed to reach statistical significance. Cmax values were comparable. It remains to be demonstrated whether these animal data can be translated into clinical findings.

Apart from the differences between amantadine salts after oral administration, it should be noted that intravenous infusions are only available as amantadine sulfate. This form of application has the following potential advantages:

1. Possibility of treatment when oral use is not possible or difficult, like in an unconscious state (e.g., TBI) or swallowing difficulties (e.g., Parkinson´s disease).
2. Faster onset of action as compared to oral administration could offer an advantage in, e.g., TBI or an akinetic crisis [175].
3. Better monitoring of the PK-PD relationship through flexible adjustment of the infusion speed.

## 9. Future Research Questions

There is some robust, though limited, evidence that amantadine is effective and safe in the treatment of consequences of TBI. The results of the largest, randomized, placebo-controlled clinical trial by Giacino et al. (2012) are further supported by a number of rather heterogenous studies employing different clinical scales and readouts in various populations of patients who had undergone TBI (see Table 1 for additional information) [131]. The current state of knowledge is reflected in several guidelines and recommendation papers. There are multiple preclinical publications existing that suggest a wide array of potential mechanisms by which amantadine may exert its beneficial effects in TBI patients. Future preclinical studies are needed to explore these mechanisms and understand how to employ them in an optimal way in clinical settings. Moreover, further clinical studies are needed to confirm and fully reveal the therapeutic potential of amantadine in patients post-TBI. There are still some important clinical questions that are yet to be answered, e.g.:

- What are the effects of amantadine in disorders of consciousness with a therapy duration of more than four weeks?
- How does amantadine work in different disorders of consciousness, especially those with non-traumatic causes?
- What is the interaction of amantadine administered in combination with other drugs (e.g., with cerebrolysin) in patients with impaired consciousness?

## 10. Conclusions

Considering the poly-pharmacology of amantadine, we believe that the potential of this compound for the treatment of TBI is not fully utilized. Amantadine may offer neuroprotective and neuroactivating benefits. The causes of TBI are diverse in terms of impact magnitude, localization, conditions of the affected person, and age. In turn, the diversity of pathological pathways may be present already in the beginning. Moreover, resulting neurodegeneration can have a severe impact on the patient’s quality of life and occurs via diverse, parallel mechanisms interacting with each other. This implies that treatment with multiple targets may show better efficacy than those with selectivity for one target. We believe that amantadine may fulfill this expectation, and in turn, well-controlled clinical studies of amantadine in TBI seem to be warranted.

In the situation when oral treatment is possible, amantadine sulfate salt may show superiority over amantadine hydrochloride due to a slower rate of absorption and, in turn, a longer duration of action connected with the decreased risk of peak-dose side effects. On the other hand, the application of amantadine as an infusion may be of particular benefit in unconscious patients with TBI by whom the oral route of administration cannot be utilized. Furthermore, intravenously administered amantadine rapidly appears in the CNS thanks to bypassing absorption from the gastrointestinal tract. Finally, parenteral administration allows for precise dose adjustment based on blood monitoring and/or patients’ physiological reactions.

The clinical practice seems to support the use of amantadine in TBI, as it is encouraged by several recommendations in different countries (e.g., in Brazil, Canada, France, Germany, USA) for practice guidelines for disorders of consciousness and TBI recovery [117,166,167,168].

## Figures and Tables

**Table 1:** Summary of clinical studies with amantadine for TBI.

Reference	Dose, Treatment Duration	Study Design	Clinical Measures	Results
[139]	50–200 mg/day BID	Case series Acute inpatient rehabilitation following brain injuries *N* = 12	Functional, neurobehavioral and cognitive status (e.g., attention, concentration, alertness, arousal, reaction time, agitation, anxiety)	Improvements in attention and concentration, alertness, arousal, processing time, psychomotor speed, mobility, vocalization, agitation, anxiety, and participation in therapy.
[136,137]	25–400 mg/day	Case series TBI *N* = 7	The Mini-Mental State Examination (MMSE), Test for Severe Impairment; Clock Drawing Test; The Hopkins Verbal Learning Test; Hopkins Attention Screening Test; The Brief Test of Attention; verbal fluency tests; The Trail Making Test; Boston Naming Test	All patients had significant frontal lobe dysfunction from TBI, and 4 were “responders” while 3 were “non-responders” to amantadine treatment, with improvements in alertness, attention, executive function, cognition, speech, behavior, mood, motivation, motor abilities and psychomotor speed, as well as less dyscontrol.
[134]	50–150 mg BID over 2 weeks	RCT, crossover TBI *N* = 10 2 weeks on AMH, 2 weeks wash out, 2 weeks on placebo	Neurobehavioural Rating Score (NRS) Orientation, memory, attention, executive Rate of patients’ cognitive recovery	Amantadine had no effect on the rate of patients’ cognitive recovery. Results limited by small sample size, heterogeneous population, acute time course, and limited study power and high drop-out rate.
[40]	200 mg/day over 6 weeks	RCT, crossover Acute TBI *N* = 35 6 weeks on AMH, 6 weeks on placebo	Agitated Behavioural Scale (ABS); MMSE; Disability Rating Scale (DRS); GOS; and Functional Independence Measure (FIM-cog) scale; Galveston Orientation and Amnesia Test (GOAT)	Significant improvements in the MMSE, DRS, GOS, and FIM cognitive scale in both groups of patients recovering from acute TBI during the first 6 weeks of the study, but only in the amantadine-treatment group during the second 6 weeks. However, the groups had similar functional levels after the study had finished. Amantadine was safe in the study population.
[142]	up to 150 mg BID	RCT, crossover Brain injuries *N* = 6	Attention and concentration, fatigue	Amantadine improved attention and concentration, and reduced fatigue.
[150]	100 mg BID to 400 mg QD	Case–control, Retrospective TBI (pediatric) *N* = 118 (amantadine *n* = 54)	Ranchos Los Amigos (RLA)	Amantadine-treated subjects had a greater improvement in their RLA level during their admission. Subjective improvements were noted in most patients administered amantadine. Side effects were minimal and resolved when treatment was reduced.
[151]	up to 150 mg/d (<10 y/o) or 200 mg/d (>10 y/o)	RCT (BUT: no placebo) TBI (pediatric subjects) *N* = 27 (amantadine *n* = 17); Only per protocol set analyzed: *N* = 13 (amantadine *n* = 9)	Cognition	Improvements with amantadine in cognitive testing when compared to age- and severity-matched TBI control patients observed in those ≤2 years post-injury. The results are limited since only per-protocol analysis was used.
[158]	200 mg BID	Retrospective Cohort Severe TBI *N* = 123 (amantadine *n* = 28)	GCS and somatosensory evoked potentials	Amantadine failed to shorten the time to emerge from coma.
[138]	400 mg/day	RCT, open label, Crossover TBI *N* = 22	Executive function	Amantadine improved performance on executive function tests, correlated with a significant increase in left prefrontal cortex glucose metabolism in the first 6 male subjects enrolled.
[144]	Not provided	Cohort TBI *N* = 124 (amantadine *n* = 47)	DRS	Amantadine significantly improved recovery
[152]	100 mg BID	RCT TBI *N* = 10 (amantadine *n* = 6)	Coma Near Coma (CNC) scale, DRS, and Western NeuroSensory Stimulation Profile	Weekly rate of change in the CNC scale, DRS, and Western NeuroSensory Stimulation Profile was significantly greater with amantadine or pramipexole than without and slowed 6 weeks after treatment termination.
[140]	200 mg BID (i.v.)	RCT, open label Closed head injury *N* = 32 (amantadine *n* = 18)	GCS, survival, biochemical parameters: glycemia, malondialdehyde (MDA; marker of lipid peroxidation), beta-carotene, total SH groups	Amantadine-treated patients had reduced MDA and increased beta-carotene (antioxidant), as well as improved survival, after only 1 week of treatment.
[170]	400 mg/day	RCT, crossover Brain injuries in pediatric population *N* = 7	CNC Scale or Coma Recovery Scale—Revised (CRS-R)	Amantadine was well tolerated, but had no significant effect on CNC Scale or CRS-R.
[131]	200 mg BID, 4 weeks	RCT, crossover Post-traumatic disorders of consciousness Patients in the vegetative state or minimally conscious state 4–16 weeks after severe TBI *N* = 184 (amantadine *n* = 87)	DRS—primary outcome measure CRS-R	Amantadine accelerated the rate of functional recovery during active treatment. The rate of improvement decreased during a 2-week wash-out period in the amantadine more than in placebo group, with no difference in DRS and CRS-R scores at 6 weeks. Amantadine did not increase the incidence of adverse effects.
[171]	100 mg BID	Case–control, Retrospective Subjects with history of head concussion *N* = 50 (amantadine *n* = 25)	Verbal memory, reaction time	After 3–4 weeks, amantadine-treated patients made significantly greater improvements in verbal memory and reaction time, as well as reported fewer persistent post-concussion symptoms, when compared to matched controls (by age, sex, and concussion history).
[154]	100 mg BID, 4 weeks	RCT TBI *N* = 76 (amantadine *n* = 38)	Neuropsychiatric Inventory—Irritability (NPI-I); Neuropsychiatric Inventory—Aggression (NPI-A)	Among patients with moderate–severe irritability (≥6 months following TBI), 4 weeks of amantadine significantly improved the frequency and severity of irritability and aggression and was safe.
[155]	100 mg BID	RCT TBI *N* = 118 (amantadine *n* = 61)	Aggression, anger	Among patients (≥6 months post-TBI) with moderate-to-severe aggression, amantadine significantly reduced aggression, with no beneficial effect on anger.
[155]	100 mg BID	RCT TBI *N* = 168 (amantadine *n* = 82)	NPI	Because of a very large placebo effect, amantadine did not significantly improve irritability (in patients with moderate–severe irritability, who suffered TBI ≥6 months prior to enrollment).
[161]	100 mg BID	Cohort, retrospective TBI *N* = 139 (amantadine *n* = 70)	Agitation, length of stay in intensive care unit (ICU)	Agitation was significantly more prevalent in the amantadine group. Patients given amantadine had longer ICU lengths of stay and received more opioids.
[160]	100 mg BID	RCT severe TBI *N* = 40 (amantadine *n* = 19)	GCS	Patients having received amantadine had a faster rate of improvement in their GCS scores during the first week of treatment. No functional differences were observed at 6-month follow-up.
[172]	100 mg BID over 4 weeks	Observational severe TBI (at 2 months orally or through enteral feeding tube)	Full Outline of Unresponsiveness (FOUR) score, DRS, GOS during 4 weeks of treatment and 2 weeks post-treatment was assessed.	Improvement in cognitive function over 4 weeks of treatment interval as shown by significant improvement on FOUR score, DRS, and GOS. Recovery speed slowed down after discontinuation of amantadine. Convulsions (adverse effect) occurred in 8 out of 50 patients (5 discontinued).
[162]	100 mg BID	RCT TBI (at least 6 months prior to enrollment, with moderate–severe irritability) *N* = 119 (amantadine *n* = 59)	Cognitive battery, irritability	No differences between groups were observed after 60 days of treatment, but the placebo responses were high. Cognitive battery baseline scores for the treatment group were higher, increasing the group’s susceptibility to ceiling effects. At day 28, the mean change for the placebo group was greater (more room for improvement?).
[159]	100 mg BID increased to 200 mg BID within 3 days	Double-blind placebo-controlled trial Acute TBI (patients admitted to the intensive care unit, ICU) *N* = 66 (amantadine *n* = 33)	GCS, GOS duration of mechanical ventilation length of hospitalization fatality at the hospital mortality in patients.	No significant differences between amantadine and placebo on the GCS, GOS, duration of mechanical ventilation and hospitalization and fatality at the hospital. Statistical differences were found on GCS and GOS in discharged and deceased patients.
[147]	200 mg/day	RCT (with parallel placebo and zolpidem groups) Acute severe TBI *N* = 66 (amantadine *n* = 22)	GCS, GOS	The improvement in GCS and GOS was non-significantly better with amantadine than with zolpidem or placebo. No clinically significant adverse events were observed.
[156]	0.7–13.5 mg/kg/d; up to 400 mg/d.	*N* = 234 children and young adults (2 mo–21 y) TBI, inpatient rehabilitation (amantadine *n* = 49 (21%) patients, 0.9–20 years)	Retrospective review of behavioral descriptions of function based on, e.g., Coma Recovery Scale-Revised (CRS-R) and post-traumatic amnesia (PTA) as measured using, e.g., Children’s Orientation and Amnesia Test	Almost half of the patients admitted with a disorder of consciousness (median age 11.6 years) were treated with amantadine Nausea/abdominal discomfort (N = 3) and agitation (N = 3) were the most commonly reported adverse effects (8 patients; 16%). None of the adverse events were reported as serious.
[148]	100 mg BID for 14 days, then 150 mg BID for 7 days, then 200 mg BID for 21 days	RCT (triple-blind, placebo-controlled Severe TBI *N* = 57 (amantadine *n* = 29)	GOS, DRS	On DRS, change from baseline was significantly (*p* = 0.015) better with amantadine (10.88 ± 5.24) than with placebo (8.04 ± 4.07). No significant difference between these groups was found for GOS.
[163]	100 mg BID for 2 days, then 150 mg BID for 2 days, then 200 mg BID until recovery of consciousness	Retrospective Severe TBI amantadine *n* =60 control *n* = 344	GCS GOS-Extended Score (GCS-ES) Length of stay Mortality Recovery of command following Days to command following	No difference between these two groups was found in terms of mortality, rates of command following, or percentage of patients with severe (3–8) Glasgow Coma Scale scores at discharge. No difference in adverse events. The amantadine group was less likely to have a favorable recovery, had a longer length of hospital stay, and a longer time to command following.

**Table 2:** Comparison of pharmacokinetic analysis of amantadine sulfate and amantadine hydrochloride given orally as suspension in CMC at equimolar doses (53.36 and 50.00 mg/kg, respectively). Symbols are means of 8 replicates per group [176].

Equimolar doses	Amantadine Sulphate	Amantadine hydrochloride	Statistical Analysis
T_½_ (h)	2.07 ± 0.62	1.67 ± 0.41	Student T-test; *p* = 0.001
t_max_ (h)	1.43 ± 1.02	0.75 ± 0.38	NS
C_max_ (ng/l)	4045 ± 689.35	3911 ± 427.11	NS
AUC 0–∞ (ng*h/mL)	22,226.88 ± 4387.05	11,690 ± 1366.33	Student T-test; *p* < 0.0001

Values are mean ± SD, N = 8 per group, NS—not significant.
